# Wilson’s Disease in Oman: A National Cohort Study of Clinical Spectrum, Diagnostic Delay, and Long-Term Outcomes

**DOI:** 10.3390/clinpract15080144

**Published:** 2025-08-03

**Authors:** Said A. Al-Busafi, Juland N. Al Julandani, Zakariya Alismaeili, Juhaina J. Al Raisi

**Affiliations:** 1 Department of Medicine, College of Medicine and Health Sciences, Sultan Qaboos University, Muscat 123, Oman; 2 College of Medicine and Health Sciences, Sultan Qaboos University, Muscat 123, Oman; 3 Department of Medicine, Nizwa Hospital, Muscat 611, Oman; 4 Internal Medicine Program, Oman Medical Specialty Board, Muscat 130, Oman

**Keywords:** Wilson’s disease, neurological presentation, familial clustering, treatment compliance, long-term outcomes, Oman

## Abstract

**Background/Objectives**: Wilson’s disease (WD) is a rare autosomal recessive disorder of copper metabolism that results in hepatic, neurological, and psychiatric manifestations. Despite being described globally, data from the Middle East remains limited. This study presents the first comprehensive national cohort analysis of WD in Oman, examining clinical features, diagnostic challenges, treatment patterns, and long-term outcomes. **Methods**: A retrospective cohort study was conducted on 36 Omani patients diagnosed with WD between 2013 and 2020 at Sultan Qaboos University Hospital using AASLD diagnostic criteria. Clinical presentation, biochemical parameters, treatment regimens, and progression-free survival were analyzed. **Results**: The median age at diagnosis was 14.5 years, with a slight female predominance (55.6%). Clinical presentation varied: 25% had hepatic symptoms, 22.2% had mixed hepatic-neurological features, and 16.7% presented with neurological symptoms alone. Asymptomatic cases identified via family screening accounted for 33.3%. Diagnostic delays were most pronounced among patients presenting with neurological symptoms. A positive family history was reported in 88.9% of cases, suggesting strong familial clustering despite a low rate of consanguinity (5.6%). Regional distribution was concentrated in Ash Sharqiyah North and Muscat. Chelation therapy with trientine or penicillamine, often combined with zinc, was the mainstay of treatment. Treatment adherence was significantly associated with improved progression-free survival (*p* = 0.012). **Conclusions**: WD in Oman is marked by heterogeneous presentations, frequent diagnostic delays, and strong familial clustering. Early detection through cascade screening and sustained treatment adherence are critical for favorable outcomes. These findings support the need for national screening policies and structured long-term care models for WD in the region.

## 1. Introduction

Wilson’s disease (WD), also known as hepatolenticular degeneration, is a rare, autosomal recessive disorder caused by mutations in the *ATP7B* gene. This gene encodes a copper-transporting P-type ATPase responsible for biliary copper excretion and the addition of copper to ceruloplasmin. When this transporter does not work properly, it leads to a buildup of toxic copper, mainly affecting the liver and central nervous system, which can be fatal if not treated [1,2].

The worldwide prevalence of WD is estimated to be approximately 1 in 30,000 to 50,000 people, though this may be underestimated in populations with high consanguinity [2]. However, data on WD prevalence in Oman and the broader Middle Eastern region remain limited. The first documented case in Oman was reported by Mirza in 1994, describing a young adult female presenting with neurological symptoms [3]. Subsequently, Joshi et al. (2002) conducted a retrospective analysis of inborn errors of metabolism in Oman and identified two children diagnosed with WD among 82 cases, leading to an estimated annual incidence of 1.6 per 100,000 live births [4]. Despite these reports, little research has since examined the disease’s clinical spectrum, genetic background, or outcomes in the Omani population.

WD presents with a broad spectrum of hepatic, neurological, psychiatric, and ophthalmological symptoms. Hepatic symptoms range from asymptomatic transaminase elevation to acute liver failure and cirrhosis, while neurological issues include dystonia, tremors, dysarthria, and cognitive impairment [5]. Psychiatric disturbances, such as depression and personality changes, further complicate the clinical picture [6]. Kayser–Fleischer rings, a key ophthalmologic marker, are often found in individuals with neurological symptoms. Diagnosis depends on a combination of clinical, biochemical, and genetic tests, including serum ceruloplasmin levels, 24 h urinary copper excretion, hepatic copper measurements, and *ATP7B* mutation analysis [7]. Misdiagnosis or delayed detection can be a major cause of morbidity and mortality [8].

Management of WD requires lifelong chelation therapy with agents such as D-penicillamine, trientine, and zinc, which either enhance urinary copper excretion or prevent intestinal copper absorption [9]. Adverse effects, poor tolerability, and non-adherence to treatment contribute to disease progression and complications. In cases of acute liver failure or decompensated cirrhosis, liver transplantation remains the only definitive cure [10].

Despite well-established diagnostic and therapeutic guidelines, there is a significant lack of regional data on WD, particularly in Oman. Previous studies have not adequately characterized the clinical patterns, treatment outcomes, and long-term prognosis of Omani patients with WD. This study represents the first national cohort analysis of WD in Oman and is among the most comprehensive from the Arabian Peninsula. To fill this gap, our research aims to describe the clinical and diagnostic features, as well as the long-term outcomes, of WD in Oman, providing insights that could influence clinical practice and future research in hepatology and neurology. By analyzing patient symptom presentation, treatment responses, and disease progression, we seek to contribute a more personalized approach to managing WD in the region.

## 2. Materials and Methods

### 2.1. Study Design and Population

This retrospective cohort study was conducted at Sultan Qaboos University Hospital, a tertiary care referral center in Muscat, Oman. We reviewed the medical records of all patients diagnosed with WD between January 2013 and December 2020 at the adult hepatology clinic. WD diagnosis was confirmed based on the clinical, biochemical, and histological criteria outlined by the American Association for the Study of Liver Diseases (AASLD) guidelines [10].

Eligible participants included patients of all ages with a confirmed diagnosis of WD. Given the global rarity of WD (estimated prevalence: 1 in 30,000) [2], even national cohorts are naturally small. Our sample represents all confirmed cases over 7 years at the largest tertiary center in Oman. Exclusion criteria were incomplete medical records or co-existing chronic liver diseases that could obscure the diagnosis of WD. Ethical approval was obtained from the Medical Research Ethics Committee of the College of Medicine and Health Sciences, Sultan Qaboos University. Due to the retrospective nature of the study and use of anonymized data, the requirement for informed consent was waived. All patient data were handled in accordance with institutional privacy and confidentiality protocols. All patients underwent diagnostic testing and confirmation of WD based on AASLD criteria prior to the initiation of anti-copper therapy.

### 2.2. Data Collection

A standardized data extraction form was used to gather demographic, clinical, and laboratory data from the electronic medical record system at SQUH. The following subsections outline the variables collected and the criteria used for classification.

### 2.3. Demographic and Family History Data

Demographic data included age at diagnosis, sex, and the presence of family history or parental consanguinity. A “positive family history of WD” was defined as having one or more first- or second-degree relatives with a confirmed diagnosis of WD, as documented in clinical records through biochemical and/or genetic testing performed either at our center or referring hospitals as part of cascade family screening.

The age at first presentation was defined as the age at which the patient first exhibited symptoms attributable to WD, as recorded in retrospective clinical records. For asymptomatic individuals diagnosed through family screening, this age was the time of their initial diagnostic testing.

### 2.4. Clinical Presentation Classification

Patients who showed no hepatic, neurological, or psychiatric symptoms at the time of diagnosis but were identified through family screening were classified as asymptomatic or presymptomatic. These individuals met diagnostic criteria for WD based on biochemical and/or genetic findings, despite lacking overt clinical signs.

Clinical presentation at diagnosis was categorized into hepatic, neurological, psychiatric, ophthalmologic, or mixed groups based on the most prominent symptoms observed at that time.

- Hepatic presentations included signs such as raised liver enzymes, jaundice, hepatomegaly, or liver failure in the absence of neurological or psychiatric features.
- Neurological presentations covered motor symptoms such as tremors, dystonia, dysarthria, or gait disturbances, without hepatic or psychiatric involvement.
- Psychiatric presentations referred to behavioral disturbances, personality changes, depression, or psychosis when they occurred alone.
- Mixed presentations were characterized by the simultaneous presence of hepatic, neurological, and/or psychiatric symptoms.
- Ophthalmologic findings, specifically the presence of Kayser–Fleischer rings, were documented separately based on slit-lamp examination results but were not used as the sole criterion for symptom classification unless accompanied by neurological involvement.

For patients identified as having “purely neurological” or “purely psychiatric” presentations, the absence of hepatic symptoms was established by the lack of clinical signs (e.g., jaundice and hepatomegaly) and normal liver biochemistry (e.g., ALT, AST, and bilirubin) documented at diagnosis. The development of hepatic symptoms during follow-up did not change the initial classification of their presentation.

### 2.5. Neuropsychiatric Severity Classification

Neuropsychiatric impairment was classified based on clinician-documented assessments at the time of diagnosis. “Moderate impairment” referred to non-disabling symptoms such as mild-to-moderate tremors, dystonia, or behavioral disturbances. “Severe impairment” included movement disorders (e.g., rigidity, mutism, or severe dystonia) or psychiatric symptoms (e.g., psychosis or severe cognitive decline) that significantly impaired daily function or required inpatient care. Because the study was retrospective, standardized rating scales were not used.

### 2.6. Biochemical and Genetic Data

Biochemical parameters recorded included serum ceruloplasmin levels, 24 h urinary copper excretion, hepatic copper concentration (when available), and *ATP7B* genetic testing results. Treatment data included the type of chelation therapy (D-penicillamine or trientine), the use of zinc therapy, treatment modifications, and documented adherence to the treatment regimen.

### 2.7. Treatment Data

Treatment options were tailored to each patient based on their clinical presentation (hepatic versus neurological), disease severity, medication availability, and tolerance to the treatment. Chelators were usually administered to symptomatic patients, while zinc was preferred for those without symptoms or during maintenance. Final decisions about treatment were at the discretion of the treating doctor.

### 2.8. Clinical Outcomes

Clinical outcomes were evaluated based on treatment response, the development of complications (e.g., cirrhosis, portal hypertension, and hepatocellular carcinoma), the need for liver transplantation, and survival status at last follow-up. These outcomes were collected retrospectively by reviewing electronic medical records, including follow-up clinic notes, laboratory results, imaging reports, and discharge summaries documented during routine care at SQUH.

### 2.9. Data Analysis

All data were entered into Microsoft Excel and analyzed with IBM SPSS Statistics version 28.0. Continuous variables were tested for normality using the Shapiro–Wilk test. Since most variables were not normally distributed, continuous data are shown as medians with interquartile ranges (IQRs), while categorical variables are summarized as frequencies and percentages. Comparisons between subgroups (e.g., hepatic vs. neurological presentations, survivors vs. deceased patients) were conducted using the Mann–Whitney U test for continuous variables and the Chi-square or Fisher’s exact test for categorical variables, as appropriate. Kaplan–Meier survival analysis was used to estimate overall survival, and differences in survival were assessed using the log-rank test. A *p*-value of <0.05 was considered statistically significant for all analyses. Missing data were handled by excluding incomplete cases from the relevant tests without imputation to preserve the integrity of the analysis.

## 3. Results

### 3.1. Epidemiological and Demographic Characteristics

A total of 36 patients with WD participated in this study (Table 1). The median age at diagnosis was 14.5 years (range: 0.8–35.0 years), whereas the median age at first presentation was slightly younger at 13.0 years, indicating a delay in diagnosis for many cases. This delay was more noticeable among patients with neurological symptoms. The group showed a slight female predominance at 55.6%. A notably high proportion of patients (88.9%) had a positive family history of WD, emphasizing the importance of family-based screening strategies. Conversely, parental consanguinity was reported in 5.6% of cases.

**Figure 1:**
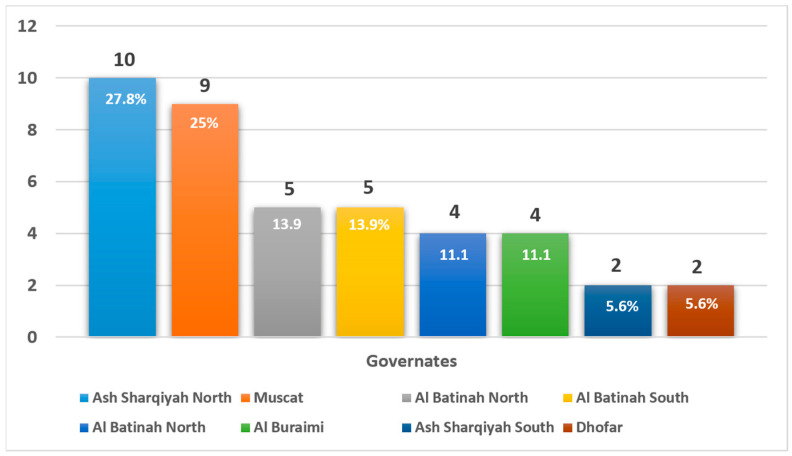
Geographic distribution of patients diagnosed with Wilson’s disease in Oman (*n* = 36).

Geographically, WD cases were unevenly distributed across Oman, with the highest concentrations in Ash Sharqiyah North (27.8%) and Muscat (25.0%) (Figure 1). These findings indicate potential regional genetic clustering and support targeted public health interventions.

Numbers above the bars indicate the number of patients from each region. These figures reflect referral patterns to the national center and do not represent a population-based prevalence. No statistical comparisons were made between regions due to these limitations.

### 3.2. Clinical Presentation and Age at Diagnosis

**Figure 2:**
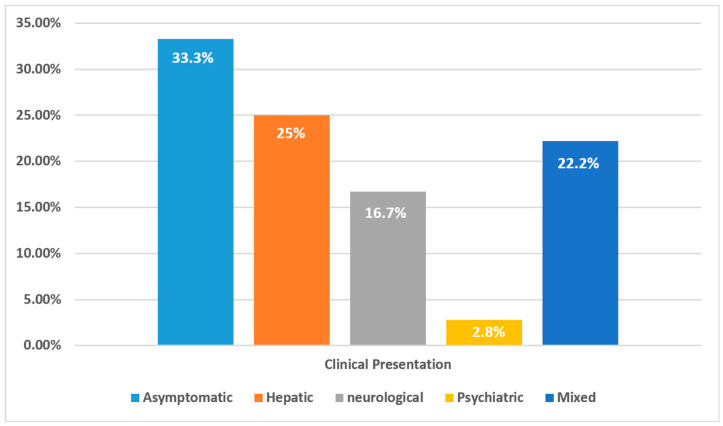
Distribution of initial clinical presentations in patients with Wilson’s disease (*n* = 36).

Table 2 outlines the clinical presentation at diagnosis. At diagnosis, one-third of the patients (33.3%, *n* = 12) were asymptomatic or presymptomatic, having been identified through family screening without obvious clinical signs. The remaining 66.7% (*n* = 24) showed symptoms, suggesting that many patients are undiagnosed until symptoms emerge. Among those with symptoms, pure hepatic symptoms were the most common (25%), followed by neurological (16.7%) and psychiatric manifestations (2.8%). Mixed symptomatology was found in 22.2% (*n* = 8) of cases, illustrating the diverse clinical spectrum of WD. Figure 2 provides a visual overview of these presentation patterns.

**Figure 3:**
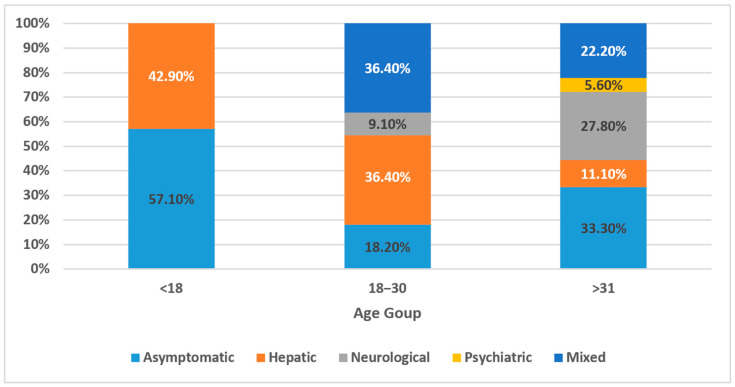
Age at diagnosis by clinical presentation in Wilson’s disease (*n* = 36).

The analysis revealed a significant association between symptom type and age at diagnosis (Kruskal–Wallis χ^2^ = 10.47, *p* = 0.033) (Table 3). Notably, asymptomatic patients were diagnosed at a significantly younger median age (11 years) compared to those with neurological symptoms (median: 24.5 years; *p* = 0.0304, Dunn’s test) (Figure 3). This confirms that neurological cases are diagnosed much later than asymptomatic or hepatic cases. These findings highlight the delays in diagnosing neurologic WD and emphasize the importance of early detection and screening, especially in at-risk families.

### 3.3. Severity at Diagnosis

#### 3.3.1. Hepatic Involvement

At diagnosis, 36.1% of patients had normal hepatic findings, 38.9% had elevated liver enzymes, 16.7% exhibited compensated liver cirrhosis, and 8.3% had progressed to decompensated cirrhosis (Table 4). These data reflect a wide spectrum of hepatic involvement at initial presentation, including advanced disease in a subset.

#### 3.3.2. Neuropsychiatric Severity

Neuropsychiatric impairment was observed in 38.9% of patients, with 19.4% classified as moderately impaired and 19.4% classified as severely impaired (Table 4). Patients with severe neuropsychiatric symptoms tended to be diagnosed at older ages, while those with normal liver and neurological function were often diagnosed earlier, usually through family screening. These results suggest a delay in diagnosis for patients with more advanced neurological symptoms (Supplementary Table S1).

### 3.4. Treatment Modalities and Outcomes

Treatment regimens varied across the cohort, with the most common medications being trientene with zinc sulfate (33.3%) and penicillamine with zinc sulfate (22.2%) (Table 5). Notably, 94.4% of patients remained in regular follow-up, and the overall mortality was 5.6% (*n* = 2) based on outcomes observed over a follow-up period ranging from 2 to 10 years, with a median duration of 6.5 years.

**Figure 4:**
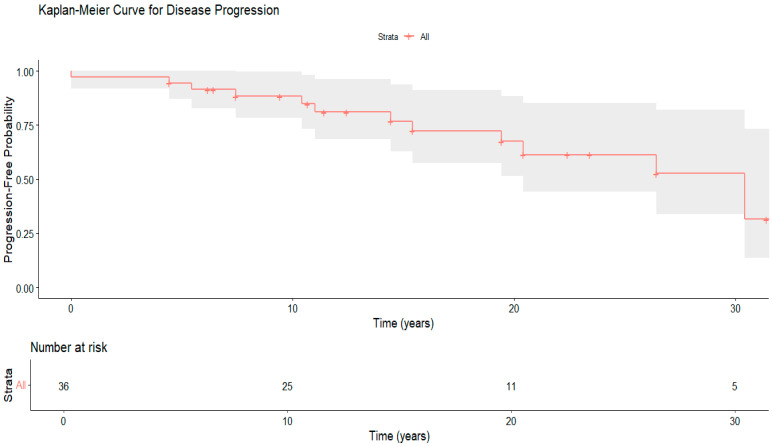
Kaplan–Meier curve depicting time to disease progression in Wilson’s disease (*n* = 36).

Kaplan–Meier survival analysis (Figure 4) demonstrates the cumulative probability of disease progression from the point of diagnosis. The median progression-free survival was 14.4 years, with an interquartile range (IQR) of 13.1 years (Table 6). This indicates a wide variability in disease progression, emphasizing the importance of personalized patient monitoring and intervention strategies. Additionally, survival decreased from 88.3% at 10 years to 61.3% at 20 years, showing a gradual increase in cumulative risk over time, highlighting the need for ongoing long-term management and follow-up strategies. The risk table below shows the number of patients at risk at each interval. This long-term outcome data offers new regional insights into the natural history of WD in Oman.

The cumulative hazard function (Nelson–Aalen; Supplementary Figure S1) shows a consistent rise in the risk of disease progression over time, with a faster increase in the second decade after diagnosis. These results support the progressive nature of WD and emphasize the need for early treatment and long-term disease monitoring.

### 3.5. Treatment Compliance and Its Impact on Disease Progression

Younger patients exhibited higher adherence rates to prescribed therapy compared to older age groups. This pattern likely results from increased parental involvement in supervising treatment. These findings indicate that adolescent and adult patients might benefit from targeted strategies to enhance adherence (Supplementary Figure S2).

**Figure 5:**
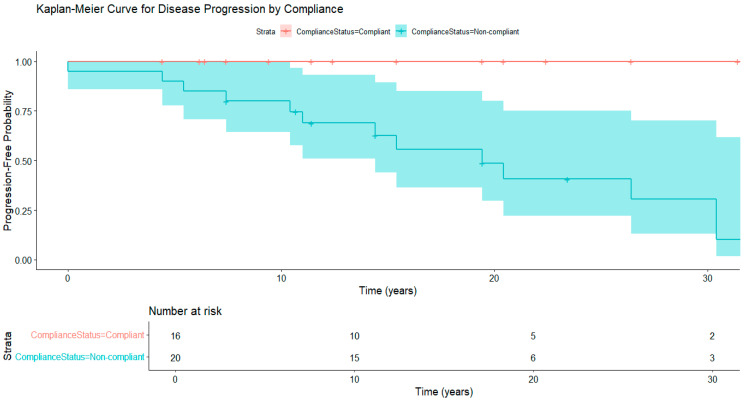
Kaplan–Meier curve of time to disease progression by treatment compliance (n = 36).

Patients who adhered to treatment had significantly longer progression-free survival times compared to those who did not (*Log-rank p* = 0.012), as shown by the Kaplan–Meier curves (Figure 5). These results emphasize the crucial role of adherence in altering the natural course of WD.

**Figure 6:**
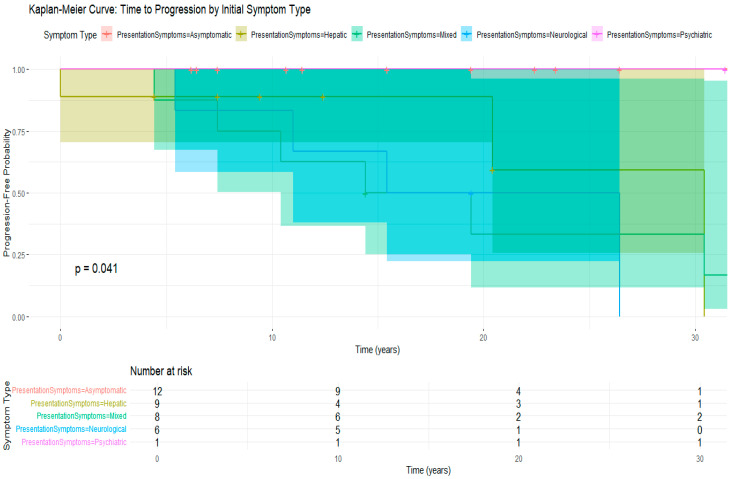
Kaplan–Meier curve for disease progression stratified by initial symptom type.

Moreover, the time to progression varied significantly depending on the initial presentation (Figure 6). Patients with neurological symptoms had the shortest progression-free survival time, followed by those in the mixed and hepatic groups (*Log-rank test: p* = 0.041). Asymptomatic individuals experienced the most favorable outcomes. These findings emphasize the importance of early detection and aggressive management in cases presenting neurological symptoms.

## 4. Discussion

This study presents the first comprehensive analysis of the clinical and epidemiological landscape of WD in Oman, offering valuable insights into diagnostic patterns, treatment adherence, disease progression, and long-term outcomes. These findings not only enhance regional understanding of WD but also provide practical guidance for improved diagnosis, patient management, and public health strategies.

A key finding is the significant delay in diagnosing patients with neurological symptoms, who were diagnosed at a median age of 24.5 years, compared to asymptomatic individuals identified through family screening, with a median age of 11 years. This diagnostic gap is among the most notable reported in the literature [11,12], suggesting a lack of recognition of WD in patients with isolated or subtle neuropsychiatric symptoms.

While many studies report the longest diagnostic delays in patients with psychiatric presentations due to symptom ambiguity [13,14], our findings identified the neurological group as experiencing the most significant delay. This may reflect regional diagnostic practices, where hepatic symptoms prompt earlier medical evaluation, and neuropsychiatric features are more likely to be misattributed to primary neurological conditions. In our cohort, the small number of isolated psychiatric cases (*n* = 1) prevented a direct comparison, but highlights the need for broader3 awareness of both neurological and psychiatric forms of WD.

Similar diagnostic delays have been described globally, particularly in settings with limited awareness or access to specialized neurological care [11,12]. The findings underscore the importance of enhancing clinical suspicion of WD among neurologists, psychiatrists, and general practitioners when encountering unexplained movement disorders, personality changes, or cognitive decline in young individuals. Prompt biochemical screening and referrals could greatly reduce delays and enhance long-term outcomes [15,16,17,18].

Our cohort exhibited an exceptionally high prevalence of positive family history (88.9%), significantly surpassing the rates typically reported in the global literature, which range from 30% to 60% [19,20]. This strong familial clustering probably reflects significant regional founder effects and increased awareness within families after a member is diagnosed. Despite a relatively low rate of consanguinity (5.6%), the findings emphasize the importance of family-based screening in enabling early diagnosis. Notably, one-third of patients were diagnosed through family screening and had no symptoms at the time of diagnosis, emphasizing the effectiveness of this method. These results align with previous studies, which have demonstrated that early detection, particularly via cascade screening, can prevent irreversible organ damage and significantly enhance long-term outcomes [16,19,20]. Our findings also support current international recommendations that advocate for cascade screening, public awareness campaigns, and genetic counseling in populations with identifiable familial clustering [10,21]. While genetic testing may be limited by cost and availability, its utility in screening high-risk families is well established and should be integrated into national healthcare strategies [12,20,22,23].

The clinical heterogeneity observed in this cohort has significant implications for both diagnosis and prognosis. Patients presenting with neurological symptoms experienced the longest diagnostic delays and had a more aggressive disease course, while hepatic cases were diagnosed earlier, although some had already progressed to cirrhosis. Specifically, 16.7% were diagnosed with compensated liver disease and 8.3% were diagnosed with decompensated liver disease at diagnosis, indicating that nearly a quarter had advanced hepatic involvement. These findings align with the literature, which links delayed recognition to worse outcomes and highlights the importance of early clinical suspicion, especially in high-risk settings [24,25]. In contrast, patients diagnosed with family screening were more likely to present early and benefit from prompt treatment. This difference emphasizes the importance of personalized, risk-based care and highlights the need to train clinicians in early detection.

Neuropsychiatric symptoms were seen in more than a third of patients, highlighting the need for a multidisciplinary diagnostic approach. Differentiating between hepatic and neurological types of WD has significant implications for prognosis and treatment. The common mistake of diagnosing neuropsychiatric symptoms as unrelated conditions reveals a gap in clinical training that can cause harmful delays. These findings support global calls for better clinician education and the development of patient-centered care pathways tailored to individual risk and presentation [26,27]. Neuropsychiatric WD continues to be underdiagnosed and often misunderstood, highlighting the need for targeted education among neurologists, psychiatrists, and general physicians to decrease diagnostic delays [28,29].

Despite a high rate of regular follow-up (94.4%), long-term progression-free survival showed a significant decline from 88.3% at 10 years to 61.3% at 20 years. Although the initial outcomes match those of global cohorts, the decrease in survival over time might be due to gaps in adherence, the shift from pediatric to adult care, or the cumulative disease burden in patients with late diagnosis [16,30]. Our findings align with well-established research, demonstrating that early and consistent treatment with chelating agents and zinc salts can slow disease progression, enhance survival rates, and prevent irreversible organ damage [16]. However, once significant hepatic or neurological damage takes place, therapeutic interventions may become less effective [18,31].

Treatment adherence proved to be a vital factor influencing outcomes. Patients who followed their treatment plans showed significantly longer progression-free survival compared to those who did not adhere to treatment. Importantly, adherence difficulties were more common among adolescents and young adults, which aligns with global evidence highlighting this group as especially vulnerable [9,16]. In our cohort, non-compliance directly led to worse outcomes, such as earlier disease progression and death. These results support previous research that highlights the importance of psychosocial support, patient education, and family involvement in maintaining long-term adherence [32,33]. Implementing targeted interventions, such as counseling, mobile health tools, and age-specific adherence programs, could help boost engagement and improve clinical outcomes.

These findings carry significant clinical and public health implications. The very high rate of familial clustering emphasizes the importance of family-based cascade screening in Oman, offering a cost-effective method for early diagnosis and the prevention of irreversible organ damage. The noticeable diagnostic delay in patients with neurological and psychiatric symptoms underscores a critical need for targeted clinician education, especially within neurology and psychiatry services, to improve awareness of WD as a potential diagnosis in young-onset neuropsychiatric syndromes. Moreover, the decrease in progression-free survival from 88.3% at 10 years to 61.3% at 20 years highlights the challenges in maintaining long-term disease management and underscores the need for structured, lifelong care models that enhance adherence and support transitions from pediatric to adult care [15,16,34]. Beyond its national importance, this study provides unique regional data to the limited research on WD from the Arabian Peninsula. Previous reports have mainly been restricted to single-center case series, making this one of the most comprehensive national-level analyses to date and the first to present 20-year Kaplan–Meier survival estimates in this population. Findings such as diagnostic delays in neurological cases, survival related to adherence, and high familial clustering offer clinically relevant insights for WD management in other low- to middle-income and resource-limited settings. Our results support the implementation of national cascade screening programs and structured long-term care models, which could also benefit other countries with similar genetic or healthcare profiles.

The study’s strengths include the use of a national cohort with detailed clinical, biochemical, and long-term outcome data, as well as the inclusion of both hepatic and neuropsychiatric phenotypes at diagnosis. The 20-year Kaplan–Meier survival data offer rare insights into the natural history of WD in a Middle Eastern population and are among the few such datasets from this region. The extended follow-up enhances our understanding of disease progression and highlights the practical challenges of maintaining long-term disease control in real-world settings. However, several limitations should be acknowledged. The retrospective design introduces potential selection and information biases, and the modest sample size (*n* = 36) limits statistical power for subgroup analyses. The absence of uniform genotypic data prevented genotype–phenotype correlations, and the adherence measurement was indirect, potentially underestimating non-compliance. Additionally, there may be underrepresentation of hepatic-only or psychiatric-only presentations due to hospital-based recruitment, and the lack of standardized neurologic severity scales restricted comparison with other cohorts. Furthermore, the study did not systematically capture treatment-related adverse events, and complications such as autoimmune reactions, nephrotoxicity, or dermatologic side effects; in particular, those associated with D-penicillamine were not documented in the available clinical records. Similarly, brain MRI data were not consistently available for review and could not be analyzed or correlated with clinical severity or outcomes, limiting the neuro-radiological interpretation of the disease spectrum. Nonetheless, the consistency of these findings with the existing literature and the regional novelty of the data strengthens the overall validity and importance of the study.

Future research should focus on prospective, multicenter studies that include Middle Eastern populations and involve larger sample sizes to verify and build upon these findings. Incorporating genetic analyses specific to populations will be crucial to identify regional mutation patterns and their clinical significance [23,35]. Although genotypic data were not available for all participants in this study, a national registry with integrated genetic testing is currently being developed to support personalized treatment approaches. Long-term studies are necessary to compare outcomes between individuals with early and delayed diagnoses and to assess their long-term quality of life [36]. Developing and validating predictive models for early WD detection, including family history and presenting symptoms, could enable earlier intervention [27]. Further research into environmental, social, and healthcare system factors impacting diagnostic delays and disease progression could help close the gaps identified in this study. Creating a national or regional WD registry with standardized genotyping, adherence monitoring, and outcome tracking will be a key step toward better care coordination. Moreover, assessing digital health tools—such as mobile apps, SMS reminders, and remote monitoring systems—might support scalable adherence strategies. Randomized trials evaluating structured transition protocols between pediatric and adult care, along with awareness campaigns to reduce diagnostic delays, would also generate valuable evidence to guide future policy and clinical practices [15,34].

## 5. Conclusions

This national cohort study offers valuable insights into the clinical spectrum, diagnostic delays, and long-term outcomes of WD in Oman. The findings show a high rate of family clustering, considerable variability in symptom presentation, particularly neurologic symptoms, and prolonged diagnostic delays that often lead to disease progression before treatment begins. These issues highlight the importance of early detection through cascade screening and enhance clinical awareness, particularly in high-risk groups. Adherence to chelation therapy was strongly linked to better clinical stability, while non-compliance was associated with poorer outcomes, underscoring the importance of patient education and structured long-term follow-up. The results support the adoption of a multidisciplinary care model that incorporates hepatology, neurology, psychiatry, and genetics, along with targeted strategies to enhance adherence, particularly during vulnerable periods such as adolescence and early adulthood. To enhance outcomes, national efforts should focus on establishing a prospective registry, incorporating genetic testing, and developing culturally appropriate adherence strategies. Future research should also investigate predictors of treatment response and the long-term effects of early versus delayed diagnosis, thereby guiding more personalized and effective care pathways for WD in Oman and providing strategies applicable to similarly resourced settings worldwide.

## Figures and Tables

**Table 1:** Epidemiological and demographic characteristics of patients with Wilson’s disease in Oman (*n* = 36).

Characteristic	Value
Age at First Presentation (years)	Median: 13 (IQR: 6.75–25.0)
Age at Diagnosis (years)	Median: 14.5 (IQR: 9.0–27.0)
Sex	
● Male	16 (44.4%)
● Female	20 (55.6%)
Family History of WD	32 (88.9%)
Parental Consanguinity	2 (5.6%)
Region of Origin	
● Ash Sharqiyah North	10 (27.8%)
● Muscat	9 (25.0%)
● Al Dakhiliyah	6 (16.7%)
● Others *	11 (30.5%)

* Others: Batinah North (13.9%), Al Batinah South (11.1%), Al Buraymi (11.1%), Ash Sharqiyah South (5.6%), and Dhofar (5.6%).

**Table 2:** Clinical presentation at diagnosis in patients with Wilson’s disease (*n* = 36).

Clinical Presentation	*n* (%)
Asymptomatic (via Family Screening)	12 (33.3%)
Pure Hepatic Symptoms	9 (25.0%)
Pure Neurological Symptoms	6 (16.7%)
Hepatic + Neurological	4 (11.1%)
Neurological + Psychiatric	2 (5.6%)
Hepatic + Neurological + Psychiatric	2 (5.6%)
Pure Psychiatric Symptoms	1 (2.8%)

**Note:** “Pure” symptom categories refer to patients presenting with only one type of manifestation (hepatic, neurological, or psychiatric). “Combined” categories indicate patients exhibiting multiple symptom domains at diagnosis. Asymptomatic patients were diagnosed through family screening and had no clinical manifestations at the time of diagnosis. Neurological and psychiatric presentations were based on clinical evaluation, neurological exam findings, and psychiatric assessment at diagnosis.

**Table 3:** Age at diagnosis by symptom type in WD patients (*n* = 36).

Presentation Symptoms	n	Mean_Age	SD_Age	Median Age at Diagnosis (IQR) *	Min_Age	Max_Age
Asymptomatic	12	11.8	7.8	11.0 **	0.8	25.0
Hepatic	9	16.0	9.4	11.0	8.0	30.0
Mixed	8	19.0	6.7	17.0	13.0	32.0
Neurological	6	25.3	7.5	24.5 **	16.0	35.0
Psychiatric	1	11.0	NA	11.0	11.0	11.0

**Note:** * The Kruskal–Wallis test showed a significant difference in age at diagnosis between groups (*p* = 0.033). ** Dunn’s post hoc test revealed that neurological cases were diagnosed significantly later than asymptomatic cases (adjusted *p* = 0.0304).

**Table 4:** Severity of hepatic and neuropsychiatric manifestations at diagnosis (*n* = 36).

Severity Level	
Hepatic Severity at Diagnosis	*n* (%)
● Normal liver function	13 (36.1%)
● Increased liver enzymes	14 (38.9%)
● Compensated liver cirrhosis	6 (16.7%)
● Decompensated liver cirrhosis	3 (8.3%)
Neuropsychiatric Severity at Diagnosis	*n* (%)
● No neuropsychiatric involvement	22 (61.1%)
● Moderately impaired	7 (19.4%)
● Severely impaired	7 (19.4%)

**Note:** The neuropsychiatric severity classification was based on clinical examination, psychiatric evaluation, and neurological assessment at diagnosis.

**Table 5:** Treatment modalities and follow-up outcomes in patients with Wilson’s disease (*n* = 36).

A. Treatment Regimens at Last Follow-Up	
Treatment Regimen	*n* (%)
Trientene + Zinc sulfate	12 (33.3%)
Penicillamine + Zinc sulfate	8 (22.2%)
Zinc sulfate only	6 (16.7%)
Penicillamine only	4 (11.1%)
All three drugs	4 (11.1%)
Trientene only	2(5.6%)
**B. Treatment and Follow-Up Outcomes**	
Outcome	*n* (%)
Regular follow-up	34 (94.4%)
Deaths	2 (5.6%)

Note: Treatment decisions were individualized based on side effect profiles, availability, and clinical response. Patients on all three drugs may have undergone regimen adjustments over time.

**Table 6:** Kaplan–Meier survival estimates in patients with Wilson’s disease (*n* = 36).

Survival Metric	Value
**Median survival time (years)**	14.4
25th percentile (Q1, years)	5.6
75th percentile (Q3, years)	18.7
Interquartile range (IQR, years)	13.1
**Estimated Survival Probability (%)**	
At 5 years	91.4%
At 10 years	88.3%
At 15 years	70.1%
At 20 years	61.3%

**Note:** Survival time refers to time from diagnosis to disease progression or death, whichever occurred first, as measured in the Kaplan–Meier model. Survival probability is defined as the proportion of patients not reaching the composite endpoint of disease progression or death by each time point.

## Data Availability

The original contributions presented in this study are included in the article. Further inquiries can be directed to the corresponding author.

## Supplementary Materials

The following supporting information can be downloaded at: https://www.mdpi.com/article/10.3390/clinpract15080144/s1, Table S1: Combined Hepatic and Neurological Severity at Diagnosis by Age in Patients with Wilson’s Disease (n = 36); Figure S1: Nelson–Aalen Cumulative Hazard Function for Disease Progression in Wilson’s Disease (n = 36); Figure S2: Treatment Compliance by Age Group in Patients with Wilson’s Disease (*n* = 36).
