# Supplementary material for: Wilson’s Disease in Oman: A National Cohort Study of Clinical Spectrum, Diagnostic Delay, and Long-Term Outcomes
Source: Clin Pract. 2025 Aug 3;15(8):144. doi: 10.3390/clinpract15080144 (PMC12384659; doi:10.3390/clinpract15080144)
Supplement: Supplementary file 1 [file clinpract-15-00144-s001.zip › clinpract-3771055-supplementary.pdf]

Supplementary Materials

Supplementary tables:

Table S1: Combined Hepatic and Neurological Severity at Diagnosis by Age in Patients with Wilson's Disease (n = 36)

Hepatic Severity	Neurological Severity	n	Mean Age at Diagnosis	SD	Median Age	Min	Max
Increased Liver Enzymes	Completely Normal	11	15.7	8.7	14.0	7.0	30.0
Completely Normal	Completely Normal	7	10.1	8.2	11.0	0.75	24.
Completely Normal	Severely Impaired	4	23.2	10.0	22.0	14.0	35.0
Completely Normal	Moderately Impaired	2	15.5	6.4	15.5	11.0	20.0
Increased Liver Enzymes	Moderately Impaired	2	26.0	8.5	26.0	20.0	32.0
Compensated Liver Cirrhosis	Completely Normal	2	9.0	1.4	9.0	8.0	10.0

Compensated Liver Cirrhosis	Moderately Impaired	2	23.0	12.7	23.0	14.0	32.0
Compensated Liver Cirrhosis	Severely Impaired	2	18.5	7.8	18.5	13.0	24.0
Decompensated Liver Cirrhosis	Completely Normal	2	18.5	9.2	18.5	12.0	25.0
Increased Liver Enzymes	Severely Impaired	1	21.0	NA	21.0	21.0	21.0
Decompensated Liver Cirrhosis	Moderately Impaired	1	21.0	NA	21.0	21.0	21.0

Note: *SD = Standard Deviation; NA = Not Applicable (only one patient in subgroup). Hepatic and neurological severities were assessed independently at diagnosis. Combined severity patterns may reflect delayed recognition of neurologically presenting WD.*

Supplementary figures:

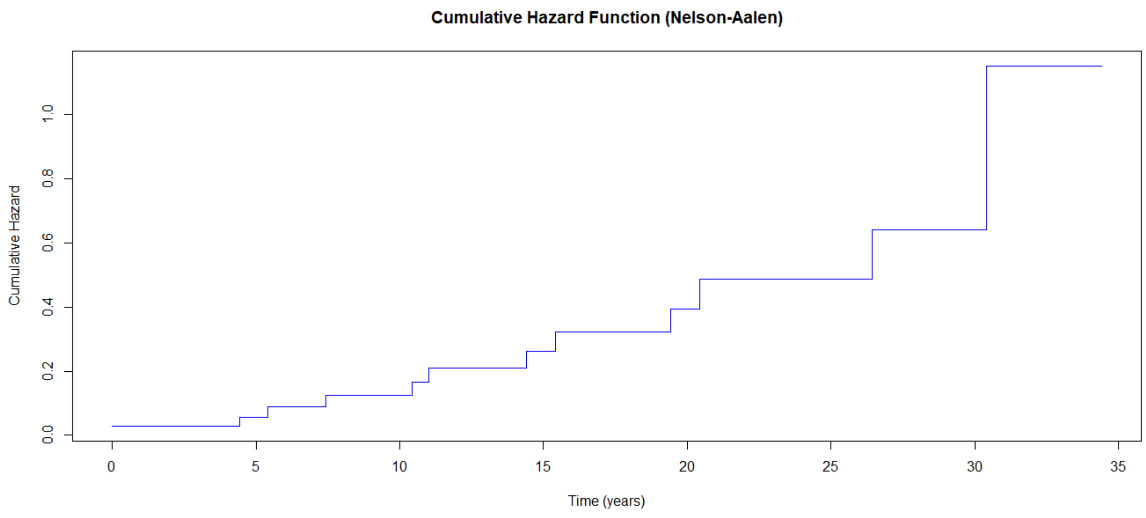

Figure S1: Nelson–Aalen Cumulative Hazard Function for Disease Progression in Wilson's Disease (n = 36)

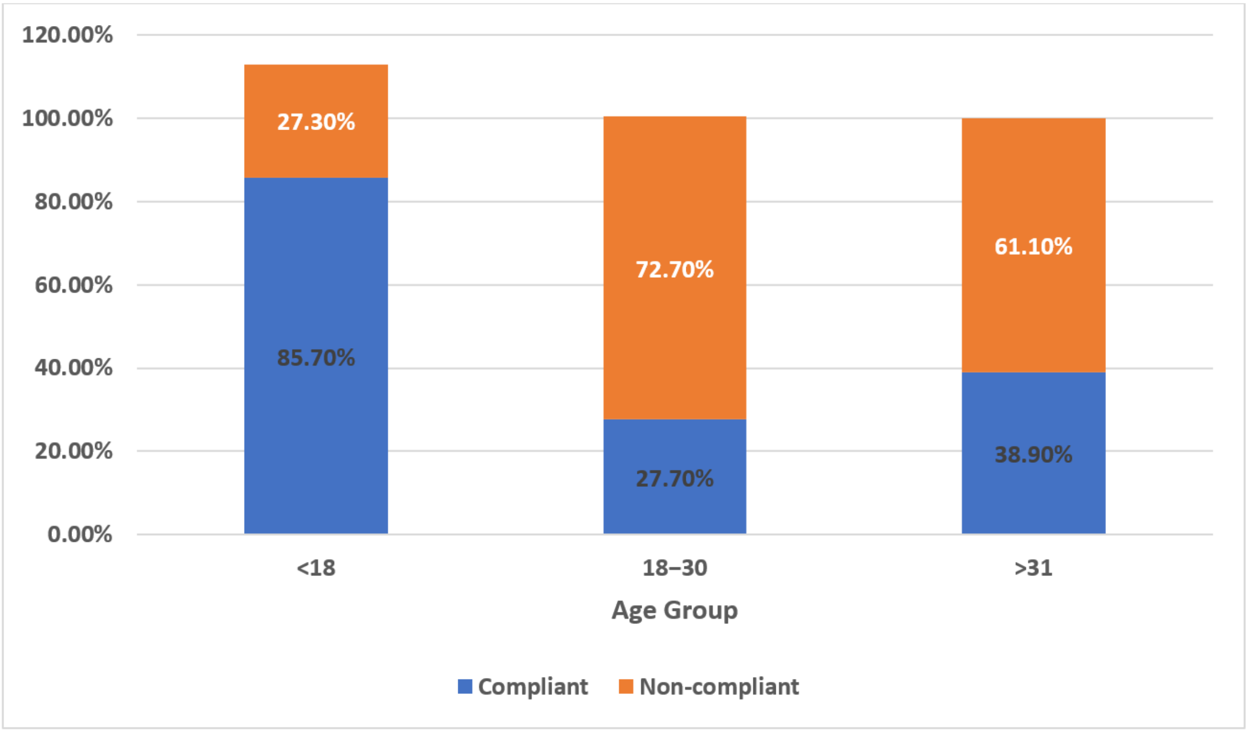

Figure S2: Treatment Compliance by Age Group in Patients with Wilson's Disease (n = 36)
